# Discriminating the Independent Influence of Cell Adhesion and Spreading Area on Stem Cell Fate Determination Using Micropatterned Surfaces

**DOI:** 10.1038/srep28708

**Published:** 2016-06-28

**Authors:** Xinlong Wang, Xiaohong Hu, Ida Dulińska-Molak, Naoki Kawazoe, Yingnan Yang, Guoping Chen

**Affiliations:** 1 International Center for Materials Nanoarchitectonics, National Institute for Materials Science, 1-1 Namiki, Tsukuba, Ibaraki 305-0044, Japan; 2 Department of Materials Science and Engineering, Graduate School of Pure and Applied Sciences, University of Tsukuba, 1-1-1 Tennodai, Tsukuba, Ibaraki 305-8571, Japan; 3 Graduate School of Life and Environmental Science, University of Tsukuba, 1-1-1 Tennodai, Tsukuba, Ibaraki 305-8571, Japan; 4 Faculty of Materials Science and Engineering, Warsaw University of Technology, Woloska 141, 02-507 Warsaw, Poland

## Abstract

Adhesion and spreading are essential processes of anchorage dependent cells involved in regulation of cell functions. Cells interact with their extracellular matrix (ECM) resulting in different degree of adhesion and spreading. However, it is not clear whether cell adhesion or cell spreading is more important for cell functions. In this study, 10 types of isotropical micropatterns that were composed of 2 μm microdots were prepared to precisely control the adhesion area and spreading area of human mesenchymal stem cells (MSCs). The respective influence of adhesion and spreading areas on stem cell functions was investigated. Adhesion area showed more significant influences on the focal adhesion formation, binding of myosin to actin fibers, cytoskeletal organization, cellular Young’s modulus, accumulation of YAP/TAZ in nuclei, osteogenic and adipogenic differentiation of MSCs than did the spreading area. The results indicated that adhesion area rather than spreading area played more important roles in regulating cell functions. This study should provide new insight of the influence of cell adhesion and spreading on cell functions and inspire the design of biomaterials to process in an effective manner for manipulation of cell functions.

As the basic behaviors of anchorage-dependent cells, adhesion and spreading play crucial roles in regulating cell functions including migration^1^,^2^,^3^,^4^, proliferation^5^,^6^ and differentiation^7^,^8^,^9^,^10^,^11^. When cells attach to a surface, they initially bind to the extracellular matrix (ECM) molecules adsorbed on the surface through integrin receptors^12^. Lateral clustering of the integrin receptors, together with other associated proteins, leads to the formation of focal adhesions (FAs) that constitute a structural link between the cytoskeleton and the ECM^13^. The FAs can respond to biochemical and biophysical stimulus by initiating a cascade of events including cytoskeleton reorganization which results in outside-in signaling activities^14^. In the meantime, the cytoskeletal force also affects the formation of FAs and is exerted to outside through the adhesion site to give feedback to their microenvironment^15^. As a consequence, the cell adhesion and spreading were manipulated by the cell/ECM interactions. Many studies have reported that the physical properties of ECM including geometry^16^,^17^, anisotropy^18^, topography^19^,^20^ and rigidity^21^,^22^ can influence the mechanosensing of the microenvironment through regulating cell adhesion and spreading. However, it is unclear whether cell adhesion or spreading is the predominant factor to influence cell functions because it has been difficult to separate the two effects by conventional cell culture using uniform surfaces.

To discriminate the influence of adhesion and spreading on cell functions, the micropatterning technology is needed because conventional ECM coating method results in parallel changes of cell adhesion and spreading areas. Several previous studies using micropatterned surfaces have reported controversial results on independent influence of adhesion and spreading areas to cell functions^23^,^24^,^25^,^26^. The controversially observed phenomena require further detailed investigation to reveal the influence of cell adhesion and spreading on cell functions. Meanwhile, how the differentiation, the most attractive point of stem cell research, is influenced by adhesion and spreading areas remains unclear. In this study, the independent influence of adhesion and spreading area on differentiation of human mesenchymal stem cells (MSCs) was investigated by using micropatterning method to precisely control cell adhesion and spreading areas. A series of micropatterns having the same size and different cell adhesion area or having different size and the same cell adhesion area were prepared by UV photolithography for cell culture. The formation of FAs and the cytoskeletal organization in the cells cultured on the micropatterns were investigated to evaluate cell adhesion and spreading state. The mechanical properties of micropatterned cells and the transduction of cytoskeletal force into nucleus were characterized to reveal the mechanism of the influence. The osteogenic and adipogenic differentiation of MSCs were investigated to show how the adhesion and spreading areas independently influenced cell fate determination.

## Results

### Preparation and characterization of micropatterns

**Figure 1:**
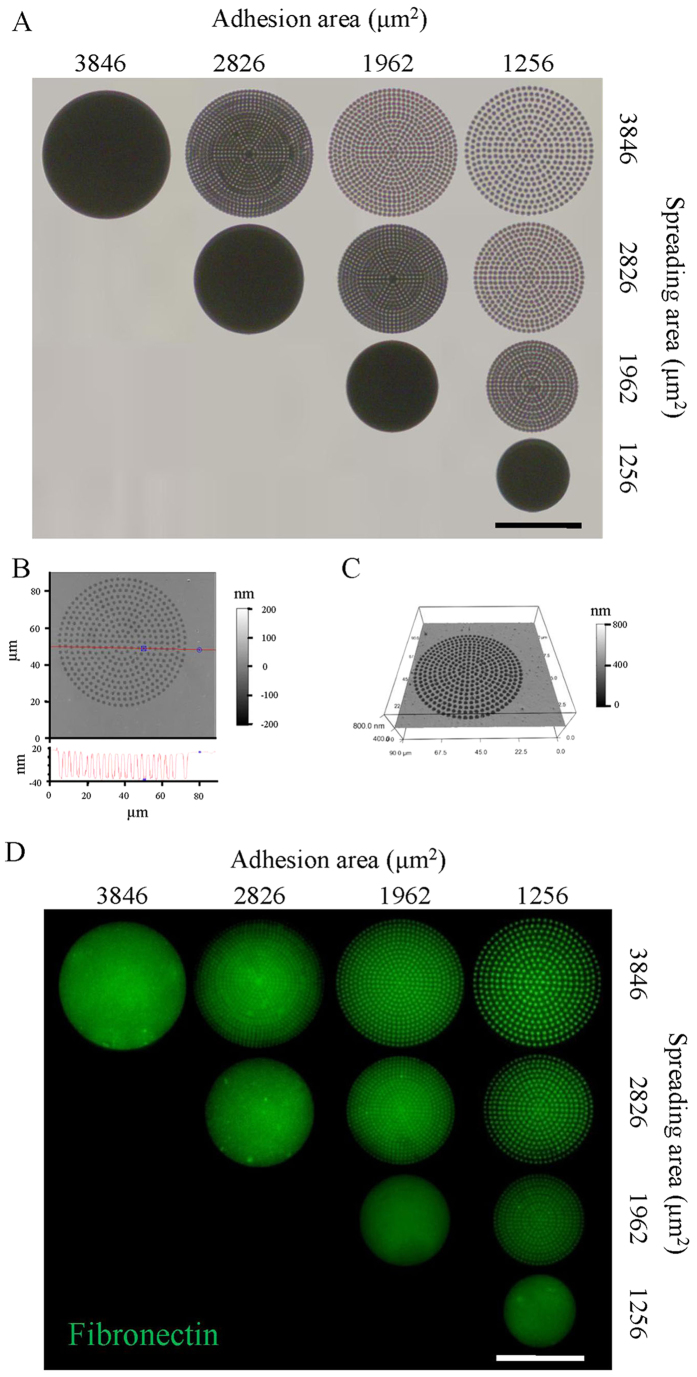
Characterization of the micropatterned TCPS surfaces. (**A**) Phase-contrast images of the photomasks designed to provide various cell adhesion and spreading areas. Scale bar: 50 μm. (**B**) The height images (up) and section view (down) of a micropattern with a diameter of 70 μm (an area of 3846 μm^2^) and the total TCPS area of 1256 μm^2^. The images were scanned by AFM. (**C**) 3D view of the micropattern shown in (**B**). (**D**) The immunofluorescence staining images of the fibronectin coated micropatterns. Fibronectin adsorbed onto the TCPS surface but not on the PVA surface. Scale bar: 50 μm.

The micropatterns were prepared by micropatterning non-adhesive PVA on cell adhesive TCPS surface (Supplementary Fig. 1). Upon UV irradiation, the photo-reactive PVA under the transparent part of the photomask was corsslinked and grafted to the TCPS surface, while those under the non-transparent microdots of the photomask remained un-reacted and were washed away by ultrasonic washing. Ten micropattern structures were designed and prepared to control cell adhesion area and cell spreading area separately (Fig. 1A). Four from the ten micropatterns were micropatterned TCPS round circles having a diameter of 70, 60, 50 and 40 μm that are shown in dark in Fig. 1A. The dark region in Fig. 1A was TCPS while white region was PVA. The other six micropatterns were composed of many TCPS microdots having a diameter of 2 μm in a round circle having a diameter of 70, 60 and 50 μm. The TCPS microdots and round circles were surrounded by PVA. Each row of the micropatterns in Fig. 1A had the same size of round circle. The four rows of micropatterns had the round circles with a diameter of 70, 60, 50 and 40 μm and corresponding area of 3846, 2826, 1962 and 1256 μm^2^, respectively. However, the total area of TCPS region (cell adhesion region) of each micropattern in the same row was different. The cells in the same row should have the same spreading area but different adhesion area. The four columns of the micropatterns had a total TCPS area of 3846, 2826, 1962 and 1256 μm^2^, respectively. The circle size of each micrpattern in the same column was different. The cells in micropatterns of the same column should have the same adhesion area but different spreading area.

The typical AFM scanning images of a circle with a diameter of 70 μm (an area of 3846 μm^2^) and the total TCPS area of 1256 μm^2^ are shown in Fig. 1B,C. The size of both large circle and small microdot were nearly the same as the designed values indicating the good controllability of the micropatterning method. The PVA layer had an average thickness of 57.8 ± 8.9 nm. After preparation of the micropatterns, fibronectin was coated on the TCPS regions. PVA is a hydrophilic polymer that can protect protein adsorption and cell adhesion^27^. Selective adsorption of fibronectin on the TCPS regions was confirmed by immunological staining of adsorbed fibronectin. The green fluorescence in Fig. 1D showed the adsorbed fibronectin on the micropatterned TCPS regions. Therefore, the fibronectin coated PVA micropatterned TCPS micropatterns with various adhesion and spreading areas were prepared using UV photolithography.

### Cell focal adhesion formation mainly regulated by adhesion area

**Figure 2:**
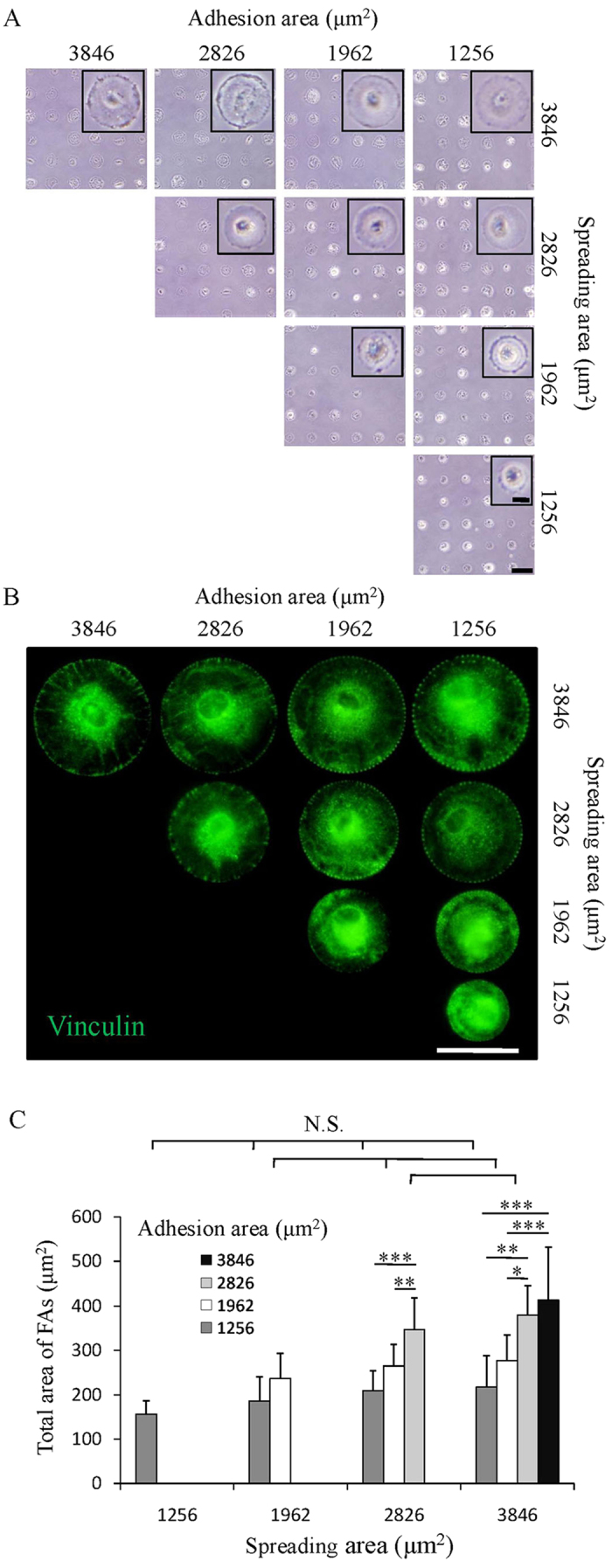
Cell adhesion and spreading on micropatterns. (**A**) Cell attachment after being cultured on micropatterned surfaces in serum-free medium for 6 h. Insert is the high magnification of representative image of attached cells. The cell morphology was well controlled by the micropatterns. Scale bar: 100 μm; insert scale bar: 20 μm. (**B**) Vinculin staining images (green) of micropatterned cells with different adhesion and spreading areas. Scale bar: 50 μm. (**C**) The total FAs area of the micropatterned cells was acquired by analyzing vinculin staining images. Data are presented as means ± SDs (n = 30). *p < 0.05, **p < 0.01, ***p < 0.001 and N.S. means no significant difference.

Human bone marrow-derived MSCs were cultured on the micropatterns. After 6 h culture in serum-free medium, MSCs adhered on the micropatterns and showed round morphology as that of the underlying micropatterns (Fig. 2A). Most of the micropatterns were occupied by single MSCs. The spreading area of MSCs was controlled by the size of each round circle of the micropatterns. The adhesion area of MSCs was controlled by the total area of fibronectin coated TCPS microdots. The MSCs on each row of micropatterns had the same spreading area but different adhesion area. The MSCs on each column of micropatterns had the same adhesion area but different spreading area. The spreading area and adhesion area of MSCs were precisely controlled by the micropatterns.

After being cultured in serum-free medium for 6 h and following serum medium for 18 h (totally 24 h), vinculin was stained to check the focal adhesions (FAs) formation of MSCs on the micropatterns (Fig. 2B).When cell spreading area was the same (each row), MSCs with large adhesion area formed more obvious FAs at the region under main cell body than did the cells with small adhesion area. When cell adhesion area was the same (each column), MSCs with large spreading area formed more obvious FAs at cell periphery region than did the cells with small spreading area. The staining images were further processed using the ImageJ software to identify the FAs level (Supplementary Figs 2 and 3). Semi-quantitative analysis of FAs revealed that the average size of FAs increased with increase of spreading areas (Supplementary Fig. 4). However, the total area of FAs slightly but not significantly increased with increase of spreading area (Fig. 2C). Increasing cell adhesion area caused significant increase of both average size and total area of FAs. The results indicated that the average size of FAs was influenced by both adhesion and spreading area, but the total area of FAs was determined mainly by the available cell adhesion area rather than the spreading area.

### Cytoskeletal organization influenced by both adhesion and spreading areas

**Figure 3:**
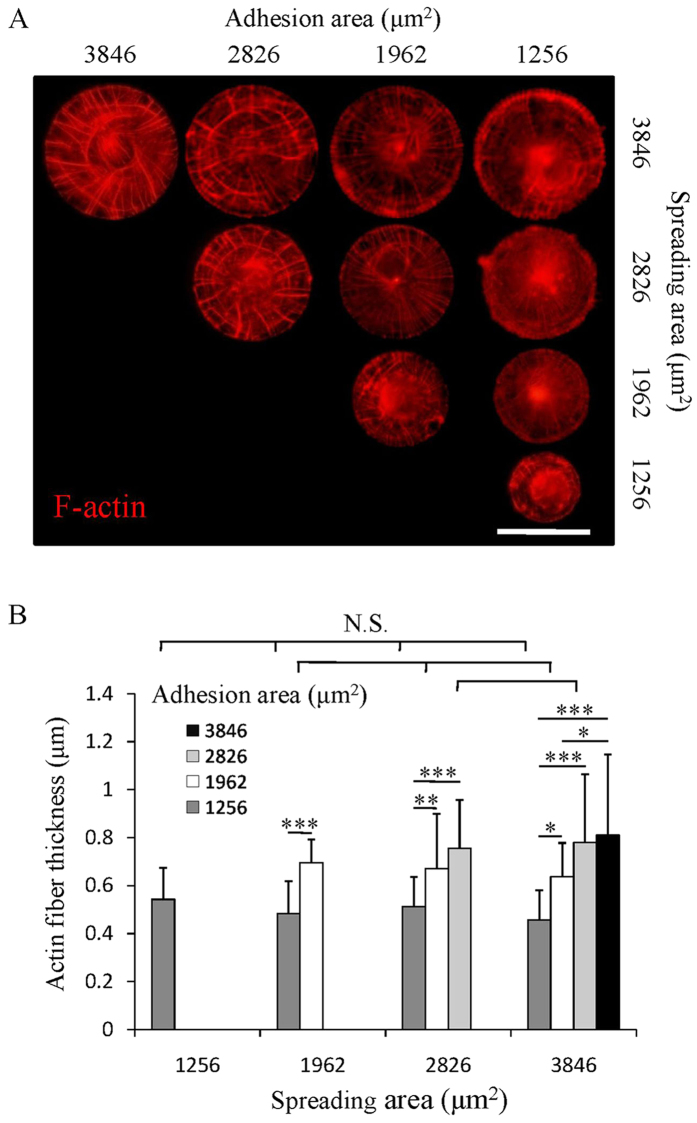
Cytoskeletal organization on micropatterns. (**A**) F-actin staining images (red) of micropatterned cells with different adhesion and spreading areas. Scale bar: 50 μm. (**B**) Actin fiber thickness of the micropatterned cells. Data are presented as means ± SDs (n = 30). *p < 0.05, **p < 0.01, ***p < 0.001 and N.S. means no significant difference.

F-actin filaments of micropatterned cells were stained to check the influence of adhesion and spreading areas on cytoskeletal organization (Fig. 3A). According to previous study, the actin stress fibers assembled in mesenchymal cells can be divided into three different subtypes including ventral stress fibers (VSFs), dorsal stress fibers (DSFs) and transverse arcs (TAs)^28^. VSFs are myosin abundant fibers that are highly contractive and are connected to the FAs at both ends. DSFs are non-contractive thin fibers that are connected to the FAs at one end. The other end of DSFs grows toward cell nuclei and weaves into actin cortex around nuclei beneath cell membrane^29^. TAs are contractive fibers that are connected to DSFs rather than to FAs. In a round micropatterned cell, VSFs and DSFs are assembled in radial direction of the circles while TAs are assembled in concentric direction of the circles^30^,^31^. In this study, the assembly of VSFs, DSFs and TAs of the micropatterned cells was determined by both the cell adhesion area and spreading area. When cell spreading area was the same (each row), cells with large adhesion area formed thicker radial fibers and more concentric fibers than did the cells with small adhesion area. When cell adhesion area was the same (each column), cells with large spreading area formed radial fibers across the whole cell body while cells with small spreading area mainly formed actin network at cell edge. The merged fluorescence images of F-actin and vinculin showed that both ends of the thick radial fibers were connected to FAs, while only one end of the thin radial fibers was connected to FAs (Supplementary Fig. 5). The results confirmed that the increase of spreading area facilitated the formation of DSFs, while the increase of adhesion area contributed to the assembly of VSFs and TAs. The thickness of F-actin fibers was analyzed to evaluate the assembly of the cytoskeleton using the ImageJ software (Supplementary Fig. 6). The thickness of F-actin fibers increased significantly with the increase of adhesion area, while did not change significantly when cell spreading area increased (Fig. 3B). Thickening of stress fibers indicated the reinforcement of the cytoskeleton which usually resulted from the increasing of cytoskeletal tension^32^.

### Cellular mechanics dominantly regulated by adhesion area

**Figure 4:**
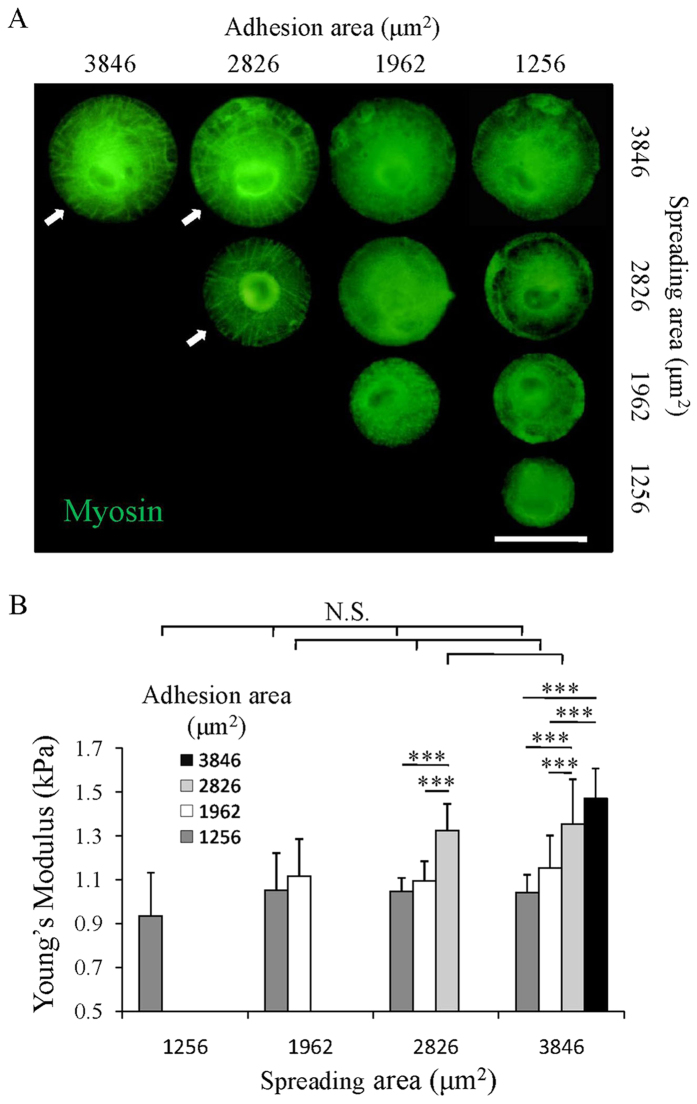
Cell mechanical state of the micropatterned cells. (**A**) Myosin (green) staining images of the micropatterned cells with different adhesion and spreading areas. Arrows indicate the micropatterned cells with a filament-like myosin structure. Scale bar: 50 μm. (**B**) The measured Young’s modulus of the micropatterned cells. Data are presented as means ± SDs (n = 20). ***p < 0.001 and N.S. means no significant difference.

Myosin as the motor protein which binds to F-actin and influences cell contractility was stained to show its distribution in MSCs cultured on the micropatterns (Fig. 4A). Although all the cells showed strong staining of myosin, the assembly of myosin was different in the cells cultured on different micropatterns. Cells with small adhesion area showed a punctuated pattern of myosin, while those with large adhesion area had filament-like myosin structures assembled in both radial and concentric directions similar to their actin structure. The merged staining images of myosin and F-actin showed a colocalization of the filament-like myosin pattern and F-actin filaments of the cells with large adhesion area (Supplementary Fig. 7). Their colocalization indicated the binding of myosin to the actin filaments. Although the cells with large spreading area and small adhesion area formed DSFs, they were depleted of filament-like myosin pattern. These results demonstrated that the myosin mainly bound to the VSFs and TAs but not to the DSFs which was in accordance with previous report^33^.

The bound myosin can generate traction force along the F-actin filaments and further influence cell mechanical state^34^. To confirm this, AFM was used to evaluate the cytoskeletal tension of MSCs cultured on the micropatterns. The average Young’s modulus of MSCs increased with increase of adhesion area (Fig. 4B). The cells with the same adhesion area had similar Young’s Modulus even they had different spreading area. The different mechanical properties should be due to the different assembly of myosin and actin filaments. Binding of myosin to the VSFs and TAs which formed in cells with large adhesion area promoted the generation of traction force and resulted in the high cytoskeletal tension of cells. Although MSCs with large spreading area and small adhesion area formed the DSFs, they still had relatively low cytoskeletal tension due to the lack of myosin binding. The results suggest that cellular mechanics was mainly determined by adhesion area rather than spreading area.

### Mechanotransduction in MSCs on micropatterns

**Figure 5:**
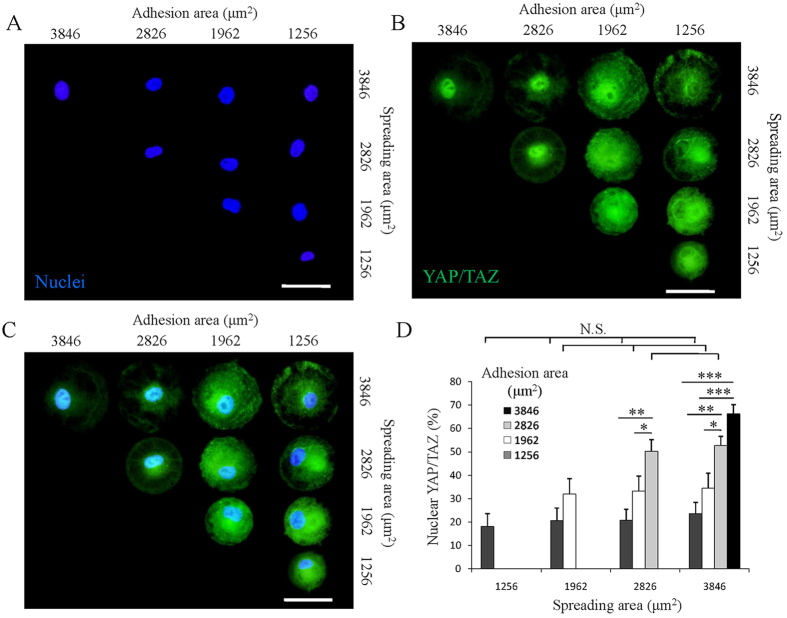
Influence of adhesion and spreading areas on mechanotransductin of the micropatterned cells. (**A**) Nuclei (blue) of single micropatterned cells were stained to show their position. Scale bar: 50 μm. (**B**) YAP/TAZ staining images (green) of the micropatterned cells. Scale bar: 50 μm. (**C**) Merged images of (**A**,**B**) to show the colocalization of the nuclei and YAP/TAZ. Scale bar: 50 μm. (**D**) The percentage of the micropatterned MSCs with nuclear YAP/TAZ. Data are presented as means ± SDs (n = 3). *p < 0.05, **p < 0.01, ***p < 0.001 and N.S. means no significant difference.

MSCs changed their cytoskeletal organization and tension depending on their adhesion and spreading areas. Since the cytoskeletal tension can be translated into the nucleus to further influence cell functions, mechanotransduction of the cells cultured on the micropatterns was investgated. Yes-associated protein (YAP) and transcriptional coactivator with PDZ-binding motif (TAZ) have been reported as sensors and mediators of the biophysical stimulus^25^. When cell encounters high cytoskeletal tension, YAP/TAZ will accumulate into cell nucleus and regulate gene transcription to influence cell functions. On the other hand, when cells cannot develop cytoskeletal tension, YAP/TAZ will exclude from nucleus and accumulate into cytoplasm. Therefore, YAP/TAZ was stained to check their localization in cells with various adhesion and spreading areas to confirm the cytoskeletal tension transduction (Fig. 5A–C). YAP/TAZ was located in the cytoplasm in the majority of cells with small adhesion area. However, increasing adhesion area facilitated the accumulation of YAP/TAZ into nucleus. By counting the number of cells with a clear colocalization of YAP/TAZ and nucleus, we confirmed that cells with the same adhesion area had similar percentage of nuclear YAP/TAZ, while the percentage increased with increase of adhesion area (Fig. 5D). This result indicated that the mechanical transduction process was mainly affected by cell adhesion area rather than spreading area.

### Influence of adhesion and spreading areas on osteogenic and adipogenic differentiation of MSCs

**Figure 6:**
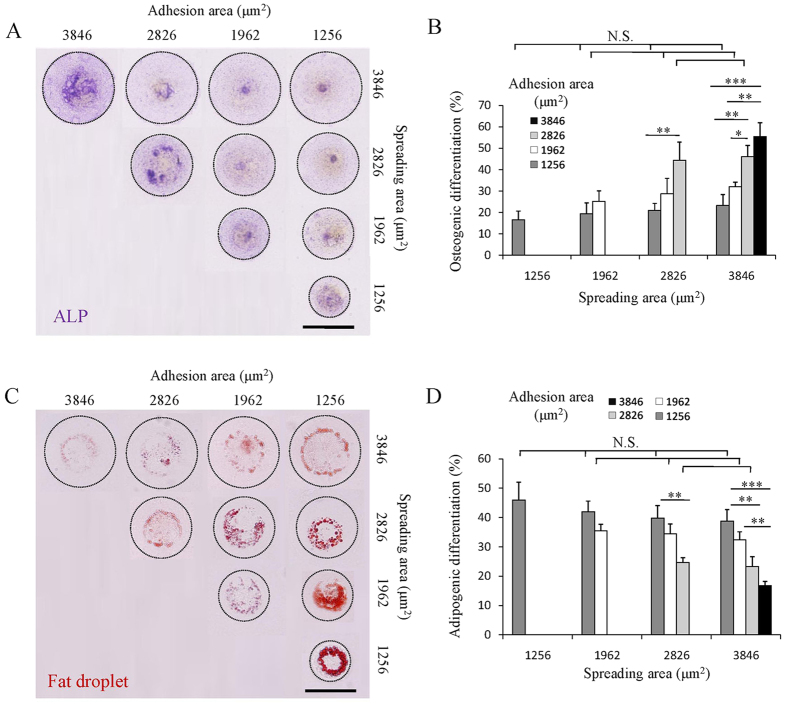
Influence of adhesion and spreading areas on osteogenic and adipogenic differentiation of MSCs. (**A**) Representative micrographs of micropatterned MSCs that are positive for ALP staining. Scale bar: 50 μm. (**B**) Percentage of ALP positive stained cells indicating the osteogenic differentiation potential of the micropatterned MSCs. (**C**) Representative micrographs of micropatterned MSCs that are positive for Oil Red O staining. Scale bar: 50 μm. (**D**) Percentage of Oil Red O positive stained cells indicating the adipogenic differentiation potential of the micropatterned MSCs. The dotted lines indicate the micropattern circles. Data are presented as means ± SDs (n = 3). *p < 0.05, **p < 0.01, ***p < 0.001 and N.S. means no significant difference.

Differentiation as one of the most important functions of stem cells was investigated to elucidate the influence of cell adhesion area and spreading area on stem cell fate determination. After being cultured on the micropatterns for 2 weeks, osteogenic differentiation was investigated by ALP staining and adipogenic differentiation was investigated by Oil Red O staining. Osteogenic differentiation of MSCs was promoted significantly by increasing cell adhesion area (Fig. 6A,B). However, increase of spreading area did not show significant influence on osteogenic differentiation. On the other hand, the adipogenic differentiation potential decreased significantly with the increase of adhesion area (Fig. 6C,D). Spreading area showed no influence on adipogenic differentiation. The results should be due to the cytoskeletal tension and activated YAP/TAZ distribution in the cells with different adhesion and spreading areas. The cells with large adhesion area showed high cytoskeletal tension and activated nuclear YAP/TAZ, and therefore had high osteogenic differentiation potential. On the other hand, cells with small adhesion area lacked cytoskeletal tension, and therefore preferred to adipogenic differentiation. The results indicated that adhesion area could regulate cytoskeletal tension and activation of nuclear YAP/TAZ and had dominant effect on the differentiation of MSCs. Adhesion area was more important than spreading area for manipulation of cell functions.

## Discussion

Manipulation of stem cell differentiation is always challenging. Efforts have been paid to elucidate the influence factors. Recently, the influence of biophysical properties of cell microenvironment including elasticity, topography, geometry, wettability, roughness and electricity on stem cell differentiation has attracted many interests^16^,^17^,^18^,^19^,^20^,^35^,^36^,^37^,^38^,^39^,^40^,^41^,^42^,^43^. Changes in most of these stimuli can cause the variation of cell adhesion and spreading which are intimately related to cell functions. Previous studies have reported that cell adhesion geometry would affect cellular force distribution^16^,^17^. However, the independent influence of adhesion area and spreading area on cell functions remains elusive. In this study, ten types of micropatterns composed of 2 μm microdots were prepared to investigate the independent influence of adhesion and spreading areas on differentiation of MSCs. The fibronectin coated micropatterns showed good capacity to precisely control the cell adhesion and spreading. Regulation of adhesion and spreading areas influenced the formation of FAs. The average size of FAs increased with increase of both adhesion area and spreading area, while the total area of FAs only increased with the increase of the adhesion area rather than spreading area (Fig. 2C and Supplementary Fig. 4). Previous studies reported that the total area and average size of FAs can be manipulated with the regulation of adhesion and spreading areas^17^,^24^,^26^. The total area and average size of FAs are related with the global cell/material adhesion strength and local maturation of FAs, respectively^44^. Our results indicated that increasing in cell spreading area would facilitate the local maturation of FAs at cell periphery region. And increasing in cell adhesion area not only promoted maturation of FAs but also reinforced the global cell/material adhesion strength.

The assembly of FAs can cause the recruitment of F-actin and direct the cytoskeletal organization^45^. Regulated by the microenvironment, cells assemble complex F-actin networks in various structures including lamellipodia, filopodia, and stress fibers^29^. The stress fibers, which are widely exhibited in micropatterned cells, can be further divided into VSFs, DSFs and TAs depending on their composition and mode of development^30^,^31^. In this study, these three types of actin stress fibers were abundant in different micropatterned cells depending on their adhesion and spreading areas. Cells with small adhesion and spreading areas had thin actin filaments only at cell edge. Increasing spreading area leaded to the assembly of DSFs and further increase of adhesion area resulted in the assembly of VSFs and TAs (Fig. 3A). The thickness of stress fibers increased with the increase of adhesion area indicated that large adhesion area facilitated the recruitment of F-actin fibers (Fig. 3B).

Previous studies classified the VSFs and TAs as myosin abundant fibers while DSFs depleted of myosin^33^. According to the staining results, although all the micropatterned cells showed strong staining intensity of myosin, myosin only associated with the VSFs and TAs and formed filament-like structure in cells with large adhesion area (Fig. 4A). Movement of myosin along actin filaments generates the traction force to regulate cell mechanical properties. This was confirmed using AFM nanoindentation. When cell spreading area was the same, cells with large adhesion area had higher cytoskeletal tension compared with those having small adhesion area. When cell adhesion area was the same, cells with various spreading areas had similar cytoskeletal tension (Fig. 4B). Previous study using micropost arrays reported a similar phenomenon that the cytoskeletal tension increased with the increase of adhesion area, and it was identical when the adhesion area was kept at the same degree regardless of their spreading area^26^.

The cytoskeletal tension regulated by cell adhesion and spreading areas can be translated into nucleus to affect gene and protein expression through mechanotransduction pathways^46^,^47^,^48^. Recent studies reported the activation of YAP/TAZ which is the transcriptional coactivators of mechanical cues involved in mechanotransduction was regulated by cytoskeletal tension and played crucial roles in stem cell differentiation^22^,^25^,^49^. In this study, the YAP/TAZ accumulated into nucleus when cells had large adhesion area, and excluded from the nucleus to cytoplasm when cells had limited adhesion area regardless of the spreading area (Fig. 5). This should be attributed to the different degree of cytoskeletal tension influenced by the binding of myosin to F-actin filaments. High cytoskeletal tension facilitated accumulation of YAP/TAZ into nucleus, while low tension facilitated accumulation of YAP/TAZ into cytoplasm.

Although previous studies have reported that parallel increase of adhesion and spreading areas would enhance osteogenic differentiation while suppresses adipogenic differentiation^50^,^51^,^52^, according to our knowledge, there is no report related to the independent influence of adhesion and spreading areas on differentiation of MSCs. Herein, the osteogenic and adipogenic differentiation of micropatterned MSCs with various adhesion and spreading areas was compared to reveal their independent influence. When cell spreading area was the same, cells with small adhesion area formed FAs at cell edge. Their cytoskeletal structure was mainly composed of radically assembled DSFs. The lack of myosin binding to DSFs resulted in low cytoskeletal tension. And the YAP/TAZ mainly distributed in cytoplasm. Therefore, cells with small adhesion area preferred to differentiate into adipocytes. While increasing in cell adhesion area reinforced the cell/material adhesion strength. Cells formed integrated actin network including VSFs, DSFs and TAs. Association of myosin with VSFs and TAs generated high cytoskeletal tension. The cytoskeletal tension stimulated accumulation of YAP/TAZ into nucleus to affect gene expression. Since the osteogenic differentiation was correlated with activation of YAP/TAZ in nucleus, cells with large adhesion area showed high potential to become osteoblasts. When cell adhesion area was the same, changing spreading area did not significantly affected stem cell fate determination. Cells with same adhesion area showed similar potential of osteogenic or adipogenic differentiation.

In conclusion, our study showed that the adhesion area rather than spreading area should play more important roles in manipulating cell functions. The adhesion area regulated the formation of FAs, cytoskeletal assembly, cell mechanical properties and mechanotransduction of micropatterned cells. Altogether, the differentiation of MSCs was determined by the available cell adhesion area rather than spreading area. Large adhesion area facilitated the osteogenic differentiation, while small adhesion area promoted the adipogenic differentiation.

## Methods

### Preperation and characterization of micropatterned surfaces

Photo-reactive azidophenyl-derived poly(vinyl alcohol) (AzPhPVA) was synthesized and micropatterned onto TCPS surface by a photolithographic method^53^. Briefly, 2 mL dicyclohexylcarbodiimide dimethyl sulfoxide (DMSO) solution (1.13 mM, Watanabe Chemical Industries, Ltd.) was added dropwise to 5 mL 4-azidobenzoic acid DMSO solution (1.13 mM, Tokyo Chemical Industry Co., Ltd.) under stirring at room temperature protected from light. After 5 min, 2 mL 4-(1-pyrrolidinyl) pyridine DMSO solution (0.113 mM, Wako Pure Chemical Industries, Ltd.) was added dropwise to the reaction mixture under stirring. After 10 min, 8 mL poly(vinyl alcohol) DMSO solution (MW 44,000, 2.26 mM in monomer units, Wako Pure Chemical Industries. Ltd.) was added dropwise to the reaction mixture under stirring. After 24 h, the reaction mixture was filtered. The filtrate was collected and dropped into methanol (Wako Pure Chemical Industries, Ltd.) to acquire the precipitate. The precipitate was dissolved in DMSO and dropped into methanol again to get the purified product. The purified photo-reactive PVA was dissolved in water to get the final product. Then the prepared photo-reactive PVA solution (200 μl, 0.3 mg/ml) was dropped onto the TCPS plate. After drying in the dark overnight, the PVA coated TCPS plate was covered by a photomask and irradiated by UV light (Funa-UV-linker FS-1500) to micropattern PVA onto TCPS plates. After ultrasonic washing, the PVA micropatterned TCPS plates were acquired (Supplementary Fig. 1).

The surface topography of micropatterns was observed by MFP-3D-BIO atomic force microscopy from Asylum Research Corporation (Santa Barbara, CA). A cantilever (spring constant: 0.06 N/m; oscillation frequency: 12–24 kHz; DNP, Bruker) with a silicon nitride tip attached to the end was used to measure the samples. The scanning was performed in Milli-Q water with a contact mode. The size and height of the micropatterns were analyzed from the acquired images. To calculate the mean values, three randomly selected micropatterns were measured.

To enhance cell adhesion and guarantee cell spreading, the sterilized micropatterns were incubated with 20 μg/ml fibronectin (Sigma-Aldrich) in NaHCO_3_ (pH = 8.4) solution for 1 h followed by exhaustive washing in NaHCO_3_ and aseptic water. To confirm the adsorption of fibronectin, the coated micropatterns were incubated with mouse anti-fibronectin (1:100, Santa Cruz Biotechnology) at 4 °C overnight. And then the micropatterns were washed and incubated with Alexa Fluor-488 labeled goat anti-mouse IgG antibody (1:800, Invitrogen) at room temperature for 1 h. The fluorescence images were observed with an Olympus BX51 microscope with a DP-70 CCD camera (Olympus, Tokyo, Japan).

### Cell culture

Human bone marrow-derived MSCs were purchased from Osiris Therapeutics, Inc. (Columbia, MA) and subcultured in MSCGM medium (MSCBM supplemented with 10% serum, 2% L-glutamine and 0.1% gentamicin sulfate amphotericin b, Lonza Group Ltd.). The fibronectin coated micropatterns were put in 6-well plates and a glass ring (inner diameter 1.5 cm) was placed over each micropattern plate. An aliquot of 3 mL serum-free medium (DMEM medium supplemented with 4500 mg/L glucose, 584 mg/L glutamine, 100 U/mL penicillin, 100 *μ*g/mL streptomycin, 0.1 mM nonessential amino acids, 0.4 mM proline, 50 mg/L ascorbic acid) was added to each well. And then 200 μL cell suspension solution (2.7 × 10^4^ cells/mL in serum-free DMEM medium) was added within the glass ring (3000 cells/cm^2^). After 6 h culture for cell attachment, the glass rings were taken out and the medium was changed to serum-containing medium for cytoskeleton development. Before medium change, cell morphology was observed by an optical microscope. After another 18 h culturein serum medium (totally 24 h), the samples were used for immunofluorescence staining and cell mechanical test.

### Immunofluorescence staining

After 24 h culture, the cells were fixed with 4% cold paraformaldehyde for 10 min. For visualization of F-actin filaments and vinculin, cells were treated with 1% Triton X-100 and 0.02% Tween-20 for 30 min. After PBS washing, the samples were blocked with 1% bovine serum albumin (BSA) in PBS for 30 min at room temperature. The samples were then incubated with the diluted mouse anti-vinculin antibodies (Merck Millipore, 1:100 in Can Get Signal solution) at 37 °C for 1.5 h followed by washing with 0.02% Tween-20 for three times. Finally the samples were incubated with Alexa Fluor-488 labeled goat anti-mouse IgG antibody (Invitrogen, 1:800) and Alexa Fluor-594 phalloidin (Invitrogen, 1:40) at 37 °C for 1 h for visualization. For myosin staining, the fixed cells were permeated with 1% Triton X-100 and blocked with 1% BSA solution for 30 min. The samples were incubated with rabbit anti-myosin IIA antibody (1:100, Sigma) at 4 °C overnight followed by PBS washing. And then the labeling was performed with Alexa Fluor-488 labeled donkey anti-rabbit IgG antibody (1:800, Invitrogen) and Alexa Fluor-594 phalloidin (Invitrogen, 1:40) at room temperature for 1 h. For YAP/TAZ staining, the fixed cells were permeated with 1% Triton X-100 and blocked with 1% BSA solution for 30 min. The samples were incubated with mouse anti-YAP/TAZ (1:50, Santa Cruz Biotechnology) at 4 °C overnight followed by PBS washing. Secondary antibody labeling was performed with Alexa Fluor-488 labeled goat anti-mouse IgG antibody (1:800, Invitrogen) at room temperature for 1 h. Nuclei were stained with DAPI. Fluorescence micrographs of the stained cells were captured using an Olympus BX51 microscope with a DP-70 CCD camera (Olympus, Tokyo, Japan).

### Image analysis

The vinculin and F-actin staining images were analyzed using an ImageJ software according to previous report^54^. Firstly, the obtained fluorescence images were converted to 16 bit images. And then the images were processed with the following commands including ‘subtracting background’, ‘enhancing local contrast’, ‘minimizing background’ and ‘adjusting brightness and contrast’. After that, the ‘Laplacian of Gaussian or Mexican Hat filter’ (available at: http://bigwww.epfl.ch/sage/soft/LoG3D) and ‘threshold’ command was run to get the final processed images (Supplementary Fig. 2). Finally, ‘analyze particles’ command was executed to calculate the focal adhesion area. The thickness of actin filaments was analyzed based on their fluorescence intensity. Gray plot profile was processed across the filaments and fitted with Gaussian fitting. The full width at half maximum was considered as the thickness of the actin filaments (Supplementary Fig. 6). The nuclear YAP percentage was analyzed from the staining images according to our previous report^55^. The fluorescence intensity of the nuclear and cytoplasm localized YAP of each micropatterned cell was acquired using the ImageJ software. When the signal in nuclear was two times higher than that in cytoplasm, the cell was determined to have nuclear localized YAP.

### Atomic force microscopy measurement

The cytoskeletal tension of living MSCs with various adhesion and spreading areas was evaluated using a commercially available MFP-3D-BIO AFM instrument in a force mode. A silicon nitride cantilever (Novascan, Ames, USA) coated with reflective gold was used for the AFM nanoindentation. At the end of the cantilever, there was a probe made of a silica glass ball with a diameter of 600 nm. An optical microscope was used to visualize the samples and the position of AFM tip. The exact spring constant of the cantilever was measured using the thermal tuning method^56^. The trigger force was set to 2 nN to avoid any damage to the cell surface. The force versus distance curves were collected at the highest region of cells at an indentation rate of 4 μm/s.

The obtained force curves were fitted to Hertz’s contact model to calculate the Young’s modulus of cells. Since the probe was a spherical ball, the parabolic model was used for calculation. The relationship between the loading force *F* and the indentation *δ* can be described in formula of:


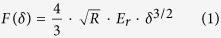


where *R* is the radius of the tip and *E*_*r*_ is the reduced Young’s modulus. In this study, the Young’s modulus of cells was calculated at 200 nm indentation depth where was reported to be abundant of actin network^57^. The reduced Young’s modulus *E*_*r*_ correlates with the Young’s modulus of sample *E*_*s*_ and is given by:


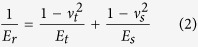


where *ν*_*t*_ and *ν*_*s*_ are the Poisson ratios of tips and samples. Since the Young’s modulus of tips material (SiO_2_) is much greater than that of living cells, equation (2) can be simplified as following:


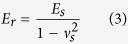


The Poisson ratio of cell is assumed to be 0.5^58^.

Ten force curves were collected from each cell. Twenty cells on each micropattern were measured to evaluate the average stiffness of the cell population. Each measurement was performed within a maximum of 1.5 hours to minimize the death of cells during the experiment.

### Osteogenic and adipogenic differentiation of micropatterned MSCs

After 1 d culture, the growth medium was replaced by osteogenic and adipogenic induction medium, respectively. The osteogenic induction medium was prepared by supplementing the DMEM medium with 1000 mg/L glucose, 584 mg/L glutamine, 100 U/mL penicillin, 100 *μ*g/mL streptomycin, 0.1 mM nonessential amino acids, 50 mg/L ascorbic acid, 10% FBS, 100 nM dexamethasone and 10 mM *β*-glycerophosphate disodium salt hydrate. The adipogenic induction medium was DMEM medium supplemented 4500 mg/L glucose, 584 mg/L glutamine, 100 U/mL penicillin, 100 *μ*g/mL streptomycin, 0.1 mM nonessential amino acids, 0.4 mM proline, 50 mg/L ascorbic acid, 10% FBS, 1 *μ*M dexamethasone, 0.5 mM methylisobutylxanthine, 10 *μ*g/mL insulin and 100 *μ*M indomethacin. The induction medium was changed every 3 d. The induction culture was continued for 2 weeks.

### Osteogenesis and adipogenesis analysis

After 2 weeks induction, the samples were fixed with 4% cold paraformaldehyde for 10 min at room temperature. The alkaline phosphatase (ALP) activity was examined by staining method to evaluate the osteogenesis of MSCs. To perform ALP staining, the fixed cells were soaked in 0.1 wt% naphthol AS-MX phosphate (Sigma) and 0.1 wt% Fast Blue RR salt (Sigma) in 56 mM 2-amino-2-methyl-1,3-propanediol (pH 9.9, Sigma) for 10 min at room temperature followed by PBS washing. The fat droplets which can be stained by Oil Red O was chosen as the maker of adipogenic differentiated MSCs. To perform Oil Red O staining, cells were firstly immersed in 60% isopropanol for 5 min and then stained with fresh Oil Red O working solution for 5 min. The Oil Red O working solution was prepared by mixing three parts 0.3% Oil Red O in isopropanol (stock solution) with two parts Milli-Q water and filtering through a 0.2 μm filter. After staining, samples were observed using an optical microscope with a DP-70 CCD camera (Olympus, Tokyo, Japan). Cells positive for ALP or fat droplets were considered as osteogenically or adipogenically differentiated. The probability of osteogenic or adipogenic differentiation of MSCs on different micropatterns was investigated by calculating the percentage of differentiated MSCs to the total cells. Three parallel experiments were carried out to calculate the means and standard deviations (SDs).

### Statistical analysis

The data of cell elasticity was presented as means ± SDs. Statistical analysis was performed using a one-way analysis of variance (ANOVA) with Tukey’s post hoc test for multiple comparisons to confirm the significant differences among samples. A value of *p* < 0.05 was considered to indicate statistically significant difference.

## Additional Information

**How to cite this article**: Wang, X. *et al*. Discriminating the Independent Influence of Cell Adhesion and Spreading Area on Stem Cell Fate Determination Using Micropatterned Surfaces. *Sci. Rep.* **6**, 28708; doi: 10.1038/srep28708 (2016).

## Supplementary Material

Supplementary Information
