# Supplementary material for: Discriminating the Independent Influence of Cell Adhesion and Spreading Area on Stem Cell Fate Determination Using Micropatterned Surfaces
Source: Sci Rep. 2016 Jun 28;6:28708. doi: 10.1038/srep28708 (PMC4923853; doi:10.1038/srep28708)
Supplement: Supplementary Information [file srep28708-s1.pdf]

Supplementary Material

Discriminating the Independent Influence of Cell Adhesion and Spreading Area on Stem Cell Fate Determination Using Micropatterned Surfaces

Xinlong Wang[1,2], Xiaohong Hu[1,3], Ida Dulińska-Molak[1,4], Naoki Kawazoe[1], Yingnan Yang[3] and Guoping Chen[1,2*]

[1]Tissue Regeneration Materials Unit, International Center for Materials Nanoarchitectonics, National Institute for Materials Science, 1-1 Namiki, Tsukuba, Ibaraki 305-0044, Japan.

[2]Graduate School of Pure and Applied Sciences, University of Tsukuba, 1-1-1 Tennodai, Tsukuba, Ibaraki 305-8571, Japan.

[3]Graduate School of Life and Environmental Science, University of Tsukuba, 1-1-1 Tennodai, Tsukuba, Ibaraki 305-8571, Japan.

[4]Faculty of Materials Science and Engineering, Warsaw University of Technology, Woloska 141, 02-507 Warsaw, Poland

Correspondence and requests for materials should be addressed to G. C. (Guoping.CHEN@nims.go.jp)

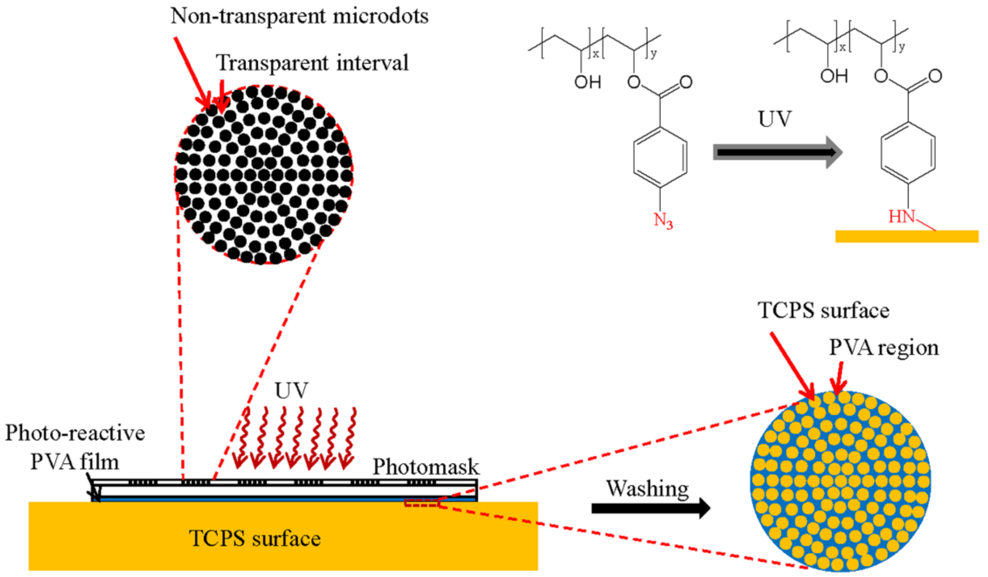

Supplementary Figure 1. Preparation scheme of the micropatterns.

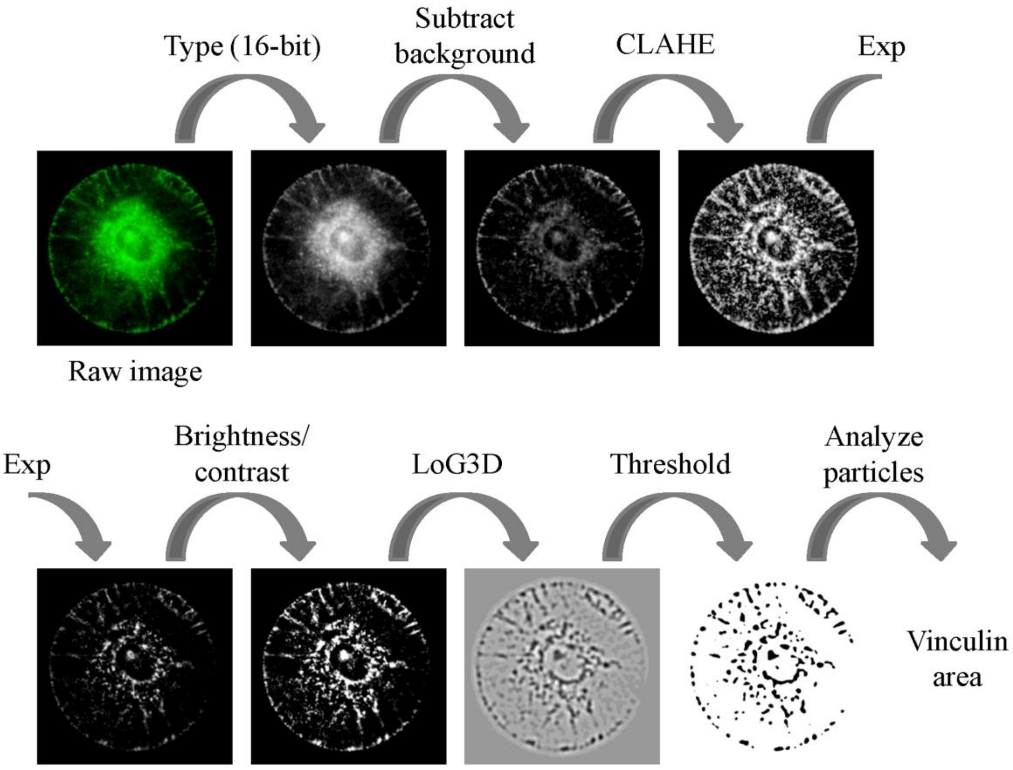

Supplementary Figure 2. Scheme of the analysis process to calculate vinculin area.

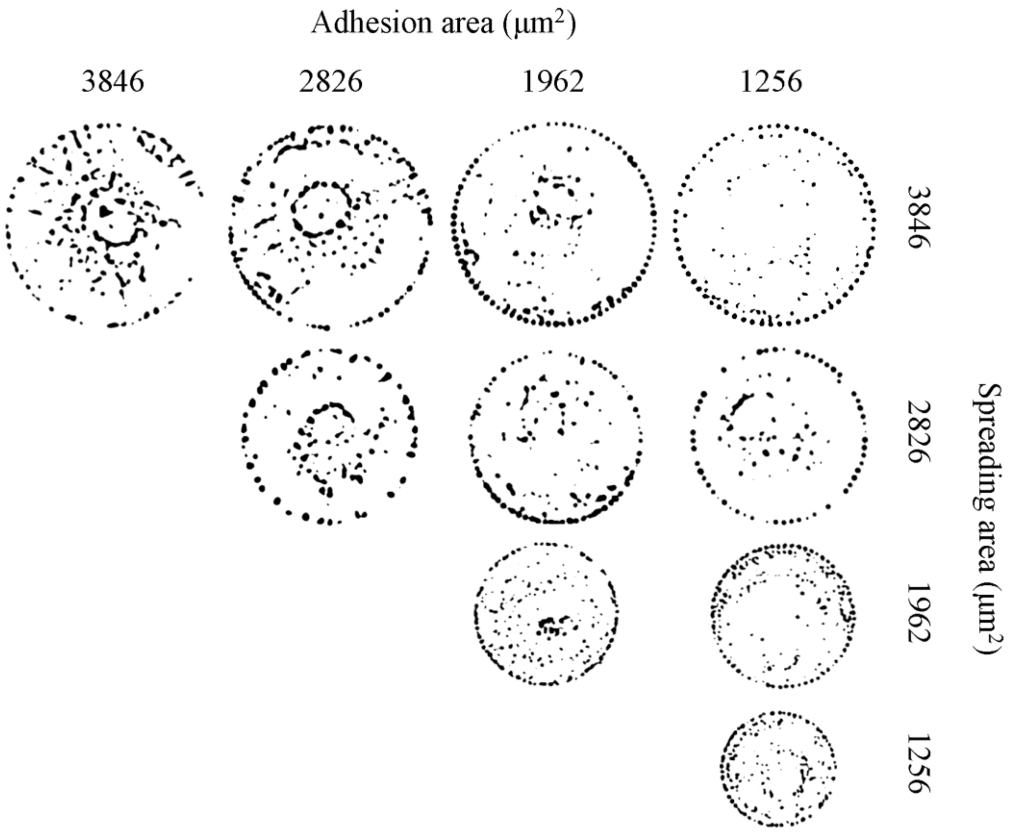

Supplementary Figure 3. The representative vinculin images of micropatterned cells with various adhesion and spreading areas after ImageJ software processing.

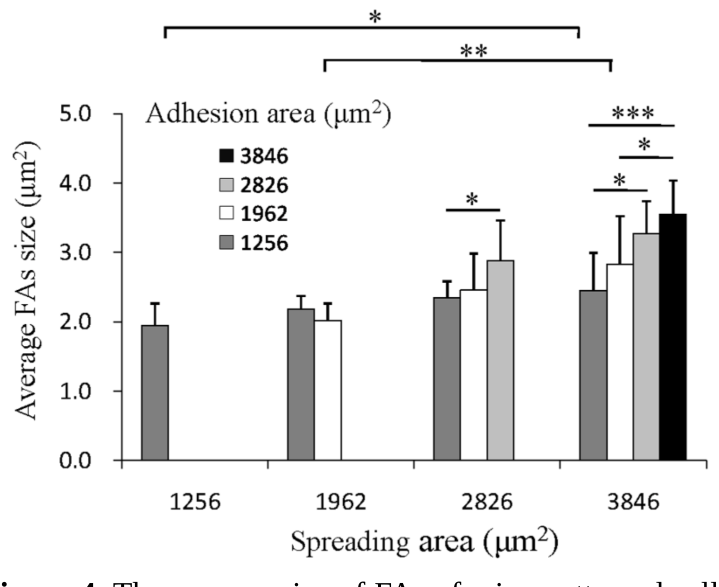

Supplementary Figure 4. The average size of FAs of micropatterned cells. *$p < 0.05$, **$p < 0.01$, ***$p < 0.001$ and unlabelled columns mean no significant difference.

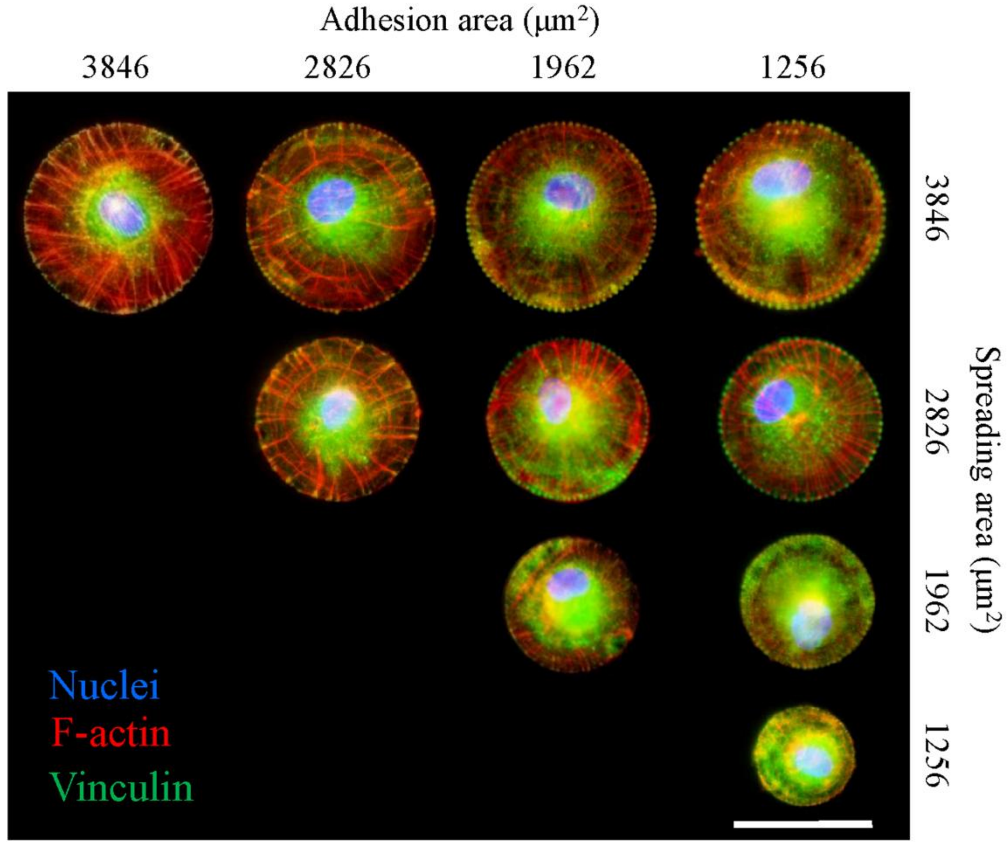

Supplementary Figure 5. The merged images of nuclei (blue), F-actin (red) and vincluin (green) of the micropatterned cells with various adhesion and spreading areas. Scale bar: 50 μm.

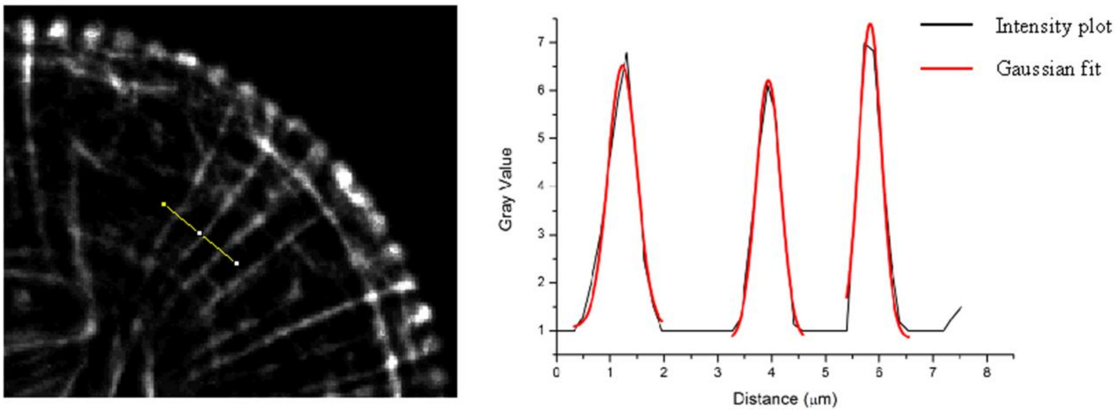

Supplementary Figure 6. Analysis of the thickness of actin fibers. Gray intensity plot of the actin fibers (right) was processed along the yellow lines (left). The intensity plot (black) was fitted to Gaussian distribution (red) to evaluate the thickness of the actin fibers.

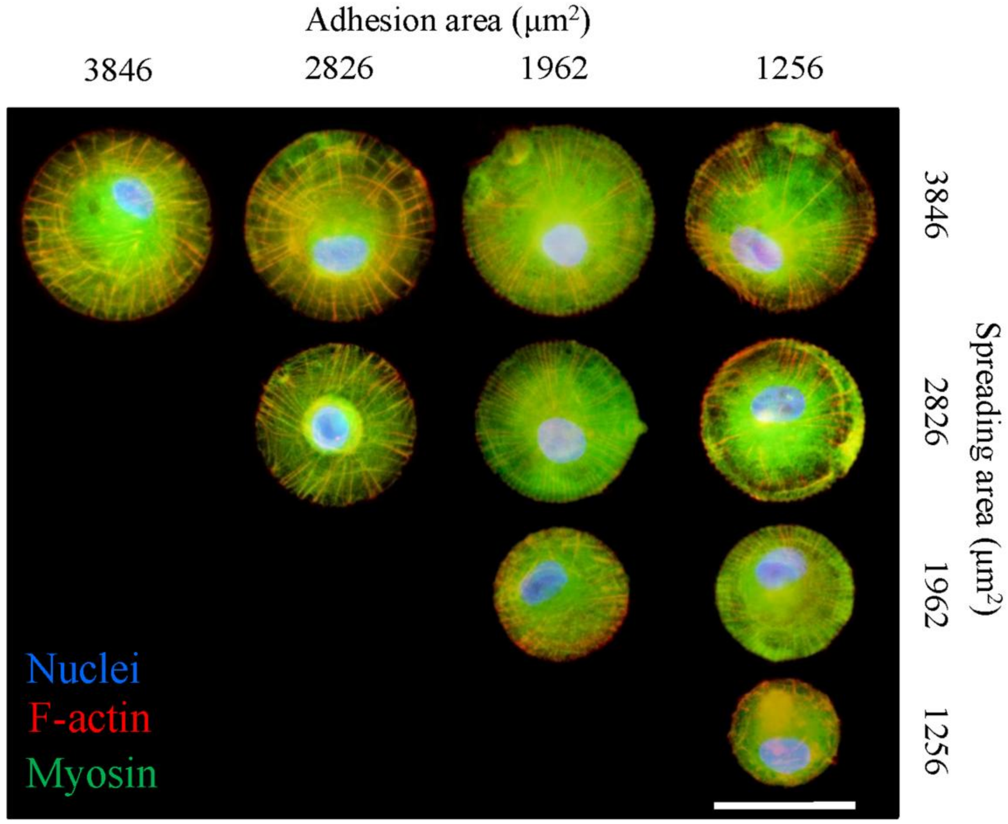

Supplementary Figure 7. The merged images of nuclei (blue), F-actin (red) and myosin (green) of the micropatterned cells with various adhesion and spreading areas. Scale bar: 50 μm.
